# Genetic variation of *Mycoplasma hyopneumoniae* from Brazilian field samples

**DOI:** 10.1186/s12866-019-1603-7

**Published:** 2019-10-28

**Authors:** Viviane Sisdelli Assao, Thalita M Scatamburlo, Elaine Nery Araujo, Marcus Rebouças Santos, Carlos Eduardo Real Pereira, Roberto Maurício Carvalho Guedes, Gustavo Costa Bressan, Juliana Lopes Rangel Fietto, Yung-Fu Chang, Maria Aparecida Scatambulo Moreira, Abelardo Silva-Júnior

**Affiliations:** 1 Federal University of Viçosa, Viçosa, Minas Gerais Brazil; 2 Federal University of Minas Gerais, Belo Horizonte, Minas Gerais Brazil; 3 Cornell University College of Veterinary Medicine, Ithaca, USA

**Keywords:** Swine, Mycoplasma hyopneumoniae, Genetic variability

## Abstract

**Background:**

Porcine enzootic pneumonia is a worldwide problem in swine production. The infected host demonstrates a respiratory disease whose etiologic agent is *Mycoplasma hyopneumoniae* (Mhp)*.* A total of 266 lung samples with *Mycoplasma-*like lesions were collected from two slaughterhouses. We analyzed the genetic profile of Mhp field samples using 16 genes that encode proteins involved in the mechanisms of bacterial pathogenesis and/or the immune responses of the host. Bioinformatic analyses were performed to classify the Mhp field samples based on their similarity according to the presence of the studied genes.

**Results:**

Our results showed variations in the frequency of the 16 studied genes among different Mhp field samples. It was also noted that samples from the same farm were genetically different from each other and samples from different regions could be genetically similar, which is evidence of the presence of different genetic profiles among the Mhp field strains that circulate in Brazilian swine herds.

**Conclusion:**

This work demonstrated the genetic diversity of several Mhp field strains based on 16 selected genes related to virulence and/or immune response in Brazil. Our findings demonstrate the difference between Mhp field strains could influence the virulence, and we hypothesize that the most frequent genes in Mhp field strains could possibly be used as vaccine candidates. Based on our results, we suspect that Mhp genetic variability may be associated with the frequency of genes among the field strains and we have demonstrated that some Mhp field samples could not have many important genes described in the literature.

## Background

*Mycoplasma hyopneumoniae* (Mhp) is the primary etiologic agent of porcine enzootic pneumonia (EP), a respiratory disease that affects swine worldwide. Swine of all ages can be affected, but animals in the growth and finishing phases are most affected [1]. EP is characterized by chronic bronchopneumonia, which is manifested by non-productive coughing [2–4]. Similar lesions can also be caused by *M. hyorhinis* [5, 6]. The available vaccines reduce the lung lesions and clinical signs in vaccinated piglets, improved daily weight gain and feed conversion ratio, but it does not prevent Mhp colonization [7, 8].

There are few studies of the virulence and genetic diversity of Mhp strains, which are used to manufacture bacterin vaccines and their relationship with Mhp field strains [9]. Charlebois et al. [10] reported molecular variability between field and vaccine strains, with less than 55% homology between the vaccine and reference strains. Some researchers have demonstrated that Mhp strains have a variety of genomic, proteomic and virulence factors [1, 11–13].

Studies of Mhp virulence factors have been based on the characterization of adherence molecules, specifically P97 adhesin [14]. Adhesins are pathogenicity factors because they recognize and bind to receptors present on the ciliated respiratory epithelium [15]. Other virulence factors and proteins, in addition to the adhesins family, may also be required for Mhp pathogenicity [16–18].

Molecular typing methods are valuable tools to differentiate strains for epidemiological investigations [10]. Researchers have investigated Mhp genetic diversity using multiple locus variable-number tandem repeat analysis (MLVA), based on two genes: P97 and P146 [19, 20]. In our work, we suggest the use of a PCR assay associated with bioinformatic tools for the analysis of genetic differences based on several Mhp genes.

In general, mycoplasmas are capable of evading the natural defenses of the ciliated respiratory epithelium through alterations of surface antigens, caused by changes in genetic mechanisms in pathogenic and non-pathogenic strains [16, 17, 21, 22]. Therefore, it is important to investigate the genetic diversity and variability of Mhp field strains to improve the vaccines and develop more specific and effective diagnostic methods.

The aim of this study was to investigate the genetic profile of Brazilian Mhp field strains in order to help the development of more effective vaccines against Mhp, as well as specific diagnostic tests for Mhp. This work provided insight into the diversity of Mhp field strains based on 16 genes using PCR assay and bioinformatic analyses. We also investigated the distribution of Mhp field strains in naturally infected farms, and their potential virulence genes.

## Results

### Mhp detection

#### PCR

Among the 266 lung samples with *Mycoplasma*-like lesions collected in both slaughterhouses in Brazil, 55.6% (148/266) were Mhp-positive by PCR, indicating the presence of MHP DNA in the samples. All lung samples were positive for endogenous control.

#### Histological analysis

**Fig. 1:**
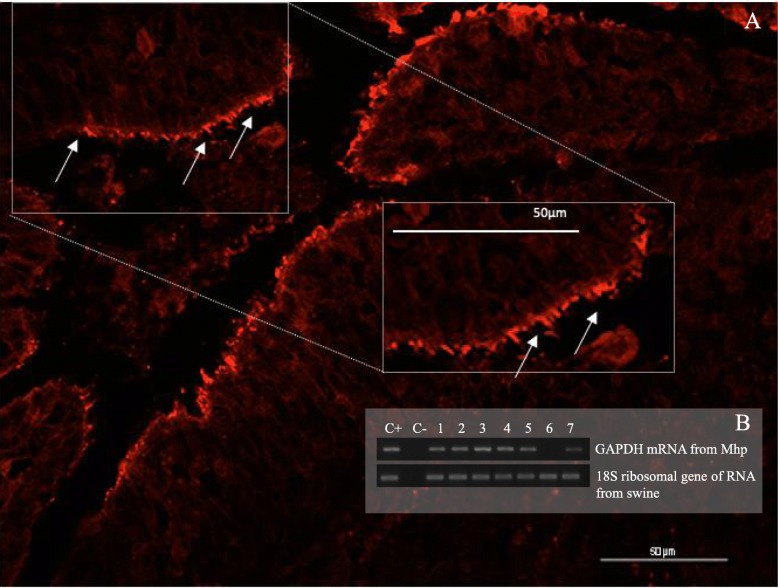
**a**- Photomicrography of FISH assay in a swine lung sample. Mhp is shown by the fluorescent markers on the surface of the epithelium and bronchiolar exudate, highlighted with white arrows. **b**- RT-PCR of *Mycoplasma hyopneumoniae* GAPDH and 18S ribosomal gene of swine. 1 to 7 are swine lung samples

Ninety-one lung samples were randomly selected to be analyzed by FISH. 70.3% (64/91) of the samples were found to be Mhp-positive with the visualization of the Mhp (Fig. 1). Among the 64 Mhp-positive samples, 24 were samples from ZM region and 40 were samples from the AP region. Only FISH-positive samples were submitted to RNA extraction using RT-PCR and demonstrated that all samples were positive for the mRNA of Mhp GAPDH.

**Fig. 2:**
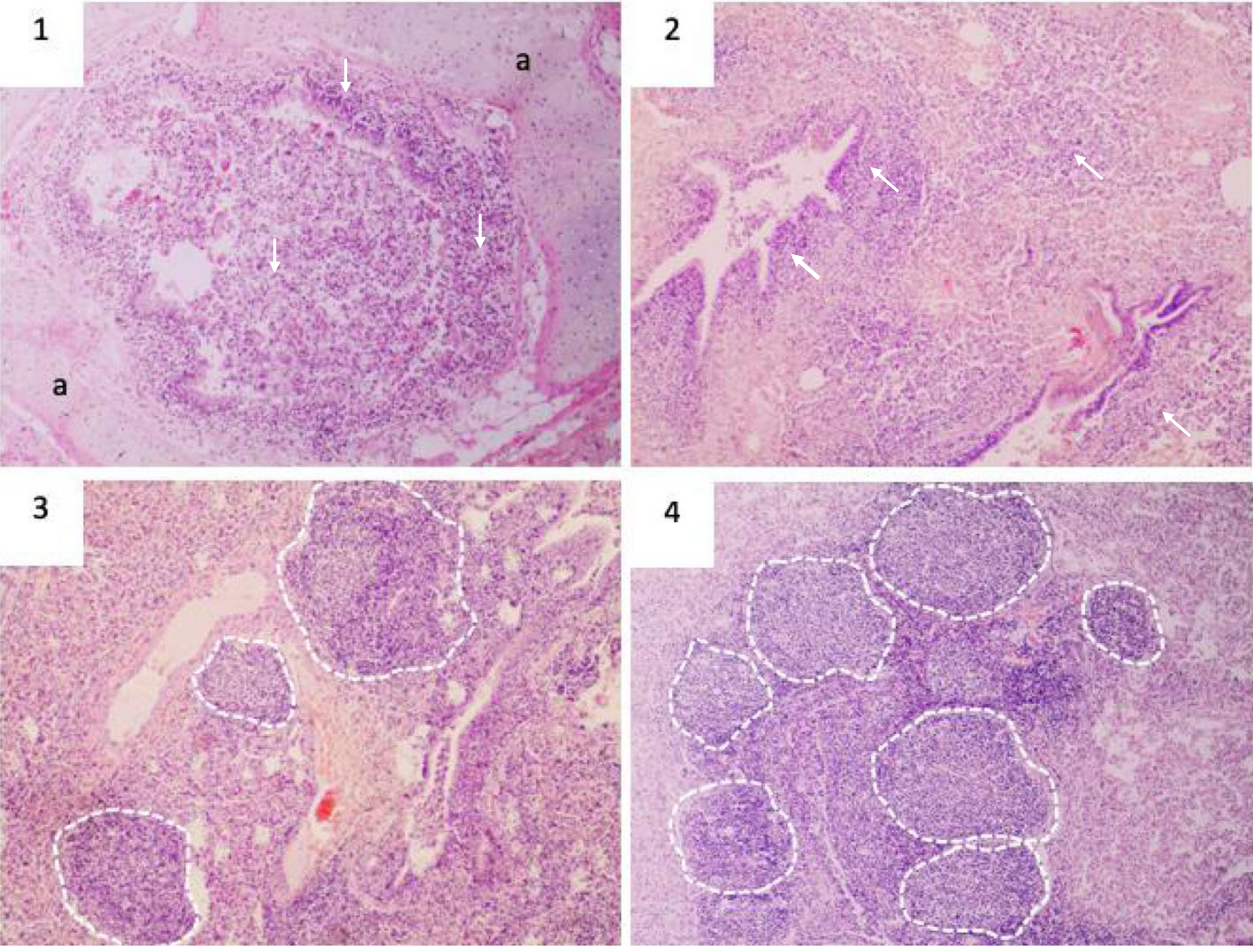
Hematoxylin and eosin staining was performed to evaluate pathological changes and lesion score. **1** – Lesion Score 1 (white arrow) moderate lymphocytic infiltrate and (a) presence of hyaline cartilage around the bronchus (100x); **2** – Lesion Score 2 (white arrow) presence of intense peribronchiolar lymphocytic infiltrate (200x); **3** – Lesion Score 3 (200x); **4** – Lesion Score 4 (100x). White circles indicate the presence of bronchus-associated lymphoid tissue (BALT) hyperplasia

**Fig. 3:**
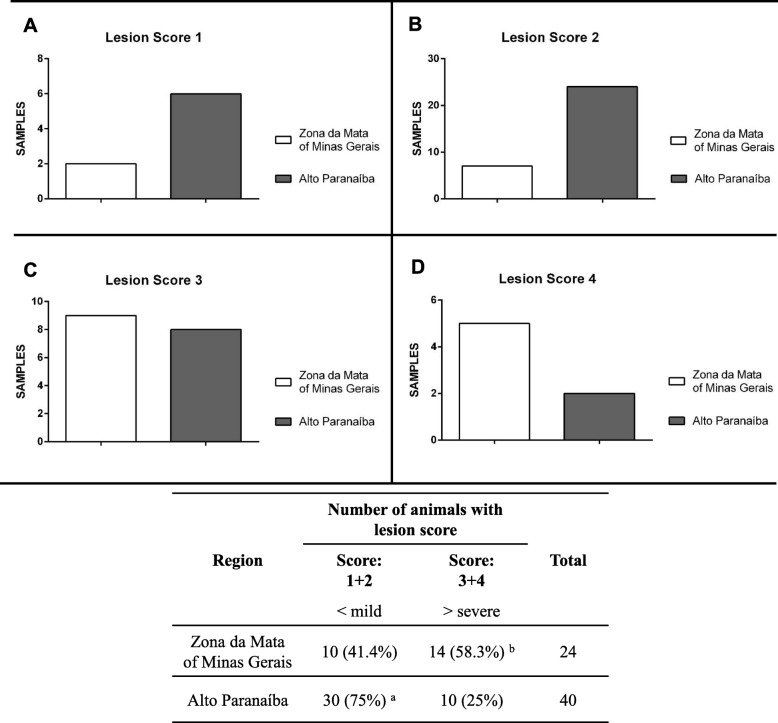
Distribution of the samples according to the lesion scores and regions. **a** represents the number of samples as lesion score 1. **b** represents the number of samples as lesion score 2. **c** represents the number of samples as lesion score 3. **d** represents the number of samples as lesion score 4. The percentage of frequency of lesion score 1 + 2 (discrete to middle) and lesion score 3 + 4 (moderate to severe). "a" denotes a statistically significant difference (*p* < 0.05; Fisher’s exact test using Yates’ correction) between the regions considering mild score, and "b" denotes a statistically significant difference (*p* < 0.05; Fisher’s exact test using Yates’ correction) between the regions considering severe scores

For histological evaluation, 64 Mhp-positive samples were selected from FISH and PCR for Mhp GAPDH mRNA. All lung samples had histological lesions characteristic of Mhp infection and different scores of lung injury (Fig. 2). According to the microscopy findings, for the ZM region: two samples were classified as Lesion Score 1, eight samples were classified as Lesion Score 2, nine samples were classified as Lesion Score 3, and five samples were classified as Lesion Score 4 (Fig. 3 and Additional file 2: Table S2). For the AP region: six samples were classified as Lesion Score 1, 24 samples were classified as Lesion Score 2, eight samples were classified as Lesion Score 3, and two samples were classified as Lesion Score 4 (Fig. 3 and Additional file 2: Table S2). The frequencies of less severe lesions (Lesion Score 1 and 2) were significantly higher in the AP samples than in the ZM samples (*p* < 0.05) (Fig. 3).

### Genetic characterization

#### PCR based characterization

Sixty-four Mhp-positive samples determined by FISH and RT-PCR, were tested for the presence of the 16 selected genes using PCR assay. Table 2 shows the gene frequency in Mhp strains, overall and separated by region. These results suggest a variation in gene frequency in Mhp field samples. The total frequency of gene presence varied, from 28.1% in Mhp field strains with the gene mhp0199 (surface protein), up to 98.4% in Mhp field strains with the genes mhp0107 (surface protein) and mhp0660 (hypothetical protein). The frequency of Mhp field strains with the gene mhp0272 (surface protein) from the ZM region was statistically higher (*p* < 0.05) compared to Mhp field strains from AP region. Moreover, the frequency of Mhp field strains with the gene mhp0418 (hypothetical protein) from AP region was statistically higher (*p* < 0.05) compared to Mhp strains from the ZM region.

**Fig. 4:**
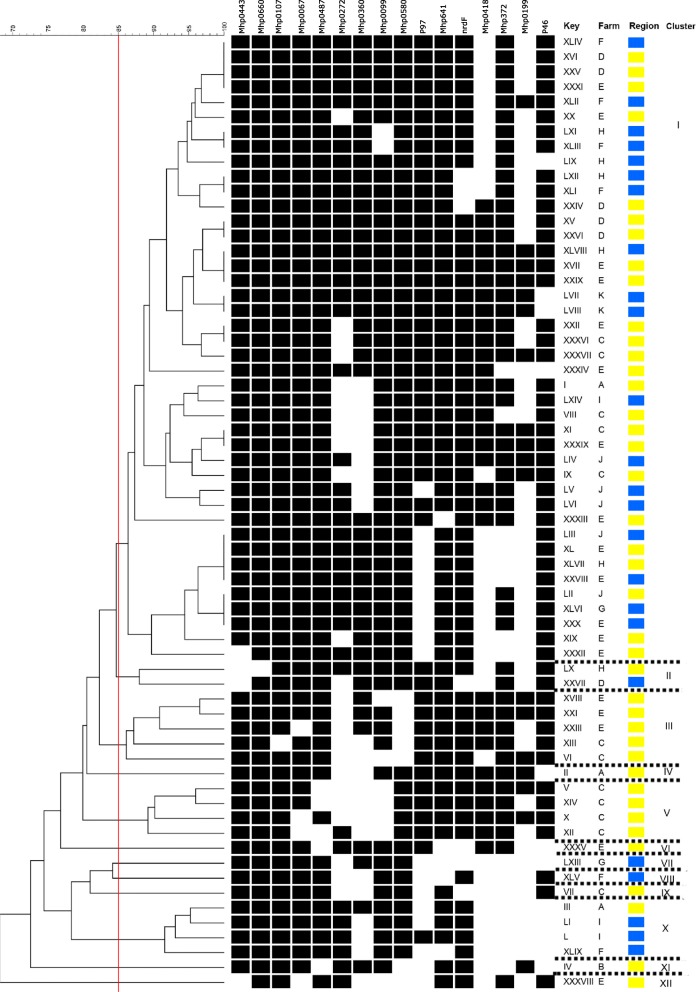
Dendrogram, created by Bionumerics software, of Mhp field strains and the 16 genes used to evaluate genetic variability, showing the 11 created clusters. Cluster analysis was performed with the following similarity parameters: Dice’s coefficient, 85% similarity cutoff value and the unweighted pair group method using arithmetic average. The number of clusters was determined with an 85% similarity cutoff value (red line). Samples are identified as roman numbers and farms are identified as letters. Regions are identified as colored squares: yellow (Alto Paranaíba) and blue (Zona da Mata of Minas Gerais). Clusters are identified with roman numerals

Sixty-four Mhp-positive samples were tested for the 16 selected genes and separated into 11 clusters according to their similarity (Fig. 4). Variation was observed in the genetic profile of the Mhp-positive samples inside the clusters. In the dendrogram, Cluster I is the biggest cluster, composed of 35 samples from eight farms from both regions. According to our results, AP samples were separated into 10 different clusters, while ZM samples were separated into five different clusters. Seven clusters were composed of single samples, five of the clusters (IV, VI, IX, X, and XI) contained samples originating from the AP region and two clusters (V and VIII) are composed of samples originating from the ZM region. The other four clusters have a larger number of samples. Cluster II contained samples from the AP region. Other clusters (I, III and VIII) are composed of samples from both regions.

#### Sanger sequencing

Some samples were randomly selected for partial sequencing, considering the molecular targets of this study. The 16 target genes had their DNA partially sequenced. A high percentage of similarity was observed between all the target genes in Mhp field strains; the percentage of similarity ranged from 98 to 100% compared to other Mhp sequences deposited into GenBank.

## Discussion

Mhp is responsible for causing respiratory disease, and its presence in swine herds could cause economic losses to the swine producers. Mhp detection is an important diagnostic of health status in the herds because if a herd has Mhp it is necessary to introduce control measures to reduce pneumonia and the associated losses. We detected Mhp DNA in lung samples with *Mycoplasma-like* lesions from Minas Gerais State. We also demonstrated that Mhp is present in animals from two important swine regions in Brazil.

We evaluated Mhp genetic diversity in swine lung samples from Minas Gerais State, Brazil and chose to detect Mhp using molecular biology because mycoplasma is one of the most fastidious slow-growing microorganisms. The genetic profiles were evaluated based on 16 genes related to virulence and/or immune response, which provides new insight into the genetic variability of Mhp field strains in an important swine-producing Brazilian state.

The DNA of 91 Mhp-positive samples was randomly selected and tested using FISH and RT-PCR assay. The FISH technique was used because it provides evidence of Mhp presence in the lung tissue. To confirm Mhp viability at a molecular level, RT-PCR was performed using the mRNA expression of an essential protein of the bacteria glycolytic pathway. These results demonstrate that Mhp cells were viable in the swine lung samples collected in the slaughterhouses.

Positive samples were evaluated for lung lesions caused by Mhp infection, at microscopic levels. The samples were classified using lesion scores based on the microscopic findings. The most frequently observed lesion scores were 1 and 2 (less severe) in AP samples, while 3 and 4 (more severe) were more frequently observed in ZM samples (Fig. 3). Fisher’s exact test using Yates’ correction (*p* < 0.05) showed that the severity of lesions found in the ZM region was statistically significantly different from the severity of lesions found in the AP region. This suggests that Mhp field strains in the ZM region could be more virulent and could be able to cause more serious lesions. It should be noted that the severity of lesions could be affected by other factors, such as management practices, vaccination, co-infection with other pathogens [4, 23].

The *mhp0272* gene encodes a P97 paralog located on the Mhp surface [24]. Our results demonstrated that *mhp0272* had a significantly higher frequency (*p* < 0.05) in the ZM samples, compared to the AP samples. This result, associated with the highest lesion score rates found in the ZM samples, suggests the more virulent potential of Mhp field strains in the ZM region could be due to the conservation of the *mhp0272* gene.

The *mhp0418* gene encodes a hypothetical protein expressed during Mhp infection [24], however, *mhp0418* was found at low frequency in this study. When we compared the detection frequency of *mhp0418* in samples from both regions studied, the gene was significantly more frequently found in the AP samples (p < 0.05) (Table 2). We also verified that Mhp field strains in the AP samples had a lower potential for pathogenicity because of less severe lung lesions in animals from this region. However, as the function of *mhp0418* is not known, it is not possible to infer about pathogenicity.

High variation in the detection of the selected genes was noted in the PCR results (Table 2), suggesting a high genetic diversity in Mhp field strains. Some of these genes encode hypothetical proteins (*mhp0443*, *mhp0660*, *mhp0487*, and *mhp0418*) whose function has not yet been clarified, but they are expressed during in vivo infection caused by Mhp [24, 25]. Some hypothetical proteins (*mhp0443*, *mhp0660*, and *mhp0487*) showed a high frequency of detection (93.8, 98.4, and 92.2%, respectively), which suggests that they could play a significant role in Mhp infection, even if their functions have not yet been determined.

Members of the P97 and P102 families are well studied because they are considered ciliary adhesins that interact with the host to facilitate Mhp pathogenesis [26]. Studies have demonstrated their potential as vaccine candidates [24, 26, 27], however, our results demonstrated that *P97, mhp0272*, and *mhp0199* are not present at a high frequency in Brazilian Mhp field samples. Only *mhp0107* had a high detection frequency. We suggest that, among the genes of P97 and P102 families studied in this work, only *mhp0107* should be used as a target for vaccine studies in this important swine producer state in Brazil.

According to the results presented in Table 2, there was a variety of detection frequencies (28.2 to 98.4%) in the genes encoding surface proteins. This could be explained because *Mycoplasma* species developed strategies to vary their surface topography to prevent detection and eradication by the host’s immune system [28, 29]. In microbial species, m exposure to new environments and the need to avoid host immune defenses contributes to selective pressures that determine which set of genes will persist [30]. This could explain the high genetic variation in the frequency of several studied genes (Table 2 and Fig. 4). The Mhp evolutionary process may have occurred through the acquisition or loss of functional genes.

A large number of genes analyzed in this study increases the characterization of the genetic profiles of Mhp field strains, since many of the Mhp field strains identified in this study we did not detect some of the studied genes; for example, samples F1 and L4 had an absence of 56.25% (9/16) of the selected genes (Table 2 and Fig. 4).

Mhp-positive field samples were separated into 11 clusters based on the similarity between the samples. The results in Fig. 4 show that a farm can have different genetic variations in Mhp field samples circulating at the same time. For example, Farm A had samples A4 and A18, which were separated into different clusters, I and XI, respectively. We also found that different regions can have Mhp field samples with similar genetic profiles. For example, Cluster VIII is composed of samples that originated from different farms in different regions, however, they were genetically similar. These findings suggest the existence of genetic diversity among Mhp field strains in Brazil*.* These results are consistent with other research, which has identified heterogeneity between Mhp strains within herds and regions in other parts of the world [10, 31–33].

In terms of Sanger sequencing (Table 3), the target genes had a high percentage of similarity with Mhp sequences deposited into GenBank, indicating that the studied genes are highly conserved in Mhp field strains in Minas Gerais State compared to Mhp strains worldwide. However, based on these results, we suggest that the genetic diversity of Mhp field strains could be affected by frequence of genes among the filed samples.

## Conclusions

This work showed the genetic diversity of several Mhp field strains based on 16 selected genes related to virulence and/or immune response in Brazil. Our findings demonstrate the difference between Mhp field strains could influence the virulence, and we hypothesize that the most frequent genes in Mhp field strains could possibly be used as vaccine candidates. Based on our results, we suspect that Mhp genetic variability may be associated with the frequence of genes among the field strains and we have demonstrated that some Mhp field samples could do not have many important genes described in the literature. The high genetic diversity among Mhp field strains should be a concern of the vaccine companies, and novel studies should be conducted to explore new vaccine candidates considering the genetic diversity in Mhp field strains.

## Methods

### Sample collection

A total of 266 lung samples with *Mycoplasma*-like lesions in the cranial or accessory lobe were collected from two slaughterhouses located in the most important *swine producing regions* in Minas Gerais, Brazil: Alto Paranaíba (AP) and Zona da Mata of Minas Gerais (ZM). These are two stand out regions, AP has 39% and ZM has 21% heads of the heard of Minas Gerais State [34]. One sample was collected from each animal. In a slaughterhouse located in the AP region, samples were collected from five farms located in the following cities: Coromandel (Farm A), Patrocínio (Farms C, D, and E) and Varjão de Minas (Farm L) (Additional file 1: Table S1). In a slaughterhouse located in ZM region, samples were collected from seven farms located in the following cities: Jequeri (Farm B), Ponte Nova (Farms F, G, and H) and Urucânia (Farms I, J, and K) (Additional file 1: Table S1). All the farms were wean-to-finish system and animals were vaccinated at weaned (one dose) for Mhp.

Each sample was divided into two pieces, one part was stored at − 80 °C to be used in molecular tests and the other fixed in formalin for histological tests. This study was registered (51/2015) and followed the recommendations of the Animal Ethics Committee of the Federal University of Viçosa.

### Mhp detection

#### Polymerase chain reaction (PCR)

Lung samples were subjected to DNA extraction using the Wizard SV Genomic DNA Purification System (Promega) following the manufacturer’s recommendations. A PCR assay was performed to detect Mhp using the primers 5′-GAGCCTTCAAGCTTCACCAGGA-3′ and 5′-TGTGTTAGTGACTTTTGCCACC-3′, which amplify a region of 649 base pairs (bp) of the 16S ribosomal RNA gene of Mhp, as described by Cai et al. [35]. As endogenous controls, primers that amplified a region of 107 bp of the 18S ribosomal gene of swine were designed: 5′-GCCTCGAAAGAGTCCTGTATTG-3′ and 5′-CTGAGAAACGGCTACCACATC-3′. PCR was performed using Go Taq Green Master Mix 2x (Promega) under the following conditions: 95 °C for 2 min, 35 cycles of 94 °C for 20 s, 60 °C for 30 s, 72 °C for 40 s, and a final extension at 72 °C for 7 min. PCR products were analyzed using 1% agarose gel electrophoresis and a transilluminator.

#### Histological analysis

The samples fixed in formalin were immersed in paraffin for staining with hematoxylin and eosin (HE) dye and fluorescence in situ hybridization (FISH). Staining was performed to identify a score for microscopic changes with a light microscope. Classification of lesion scores was performed semi-quantitatively based on Hansen et al. [36]. The lung fragments received the following classifications according to microscopic changes: Lesion Score 1 (discrete peribronchial, peribronchiolar and perivascular diffuse lymphocytic infiltrate, with discrete hyperplasia of type II pneumocytes); Lesion Score 2 (moderate peribronchial, peribronchiolar and perivascular diffuse lymphocytic infiltrate, with moderate hyperplasia of type II pneumocytes and discrete pulmonary bronchus-associated lymphoid tissue (BALT) hyperplasia); Lesion Score 3 (intense peribronchial, peribronchiolar and perivascular diffuse lymphocytic infiltrate, with hyperplasia of type II pneumocytes and moderate BALT hyperplasia); and Lesion Score 4 (intense and diffuse BALT hyperplasia).

Samples were randomly selected to detect and localize Mhp using the FISH technique. The probe included a cyanine fluorochrome, targeting the 16S ribosomal RNA gene of Mhp (5′ CCG TCA AGA CTA GAG CAT 3′). Sections of 4 μm were dewaxed in the oven at 65 °C, immersed in xylene and hydrated in several solutions of decreasing alcohols concentration and afterward, in distilled water for 5 min. The sections were incubated at 37 °C for 16 h with 100 mL of hybridization buffer (100 mM Tris pH 7.2; 0.9 M NaCl; 0.1% sodium dodecyl sulfate) and 500 ng of the probe; the entire procedure was performed in the absence of light. Slides were washed with hybridization buffer and wash buffer and, in sequence, preheated at 37 °C. Slides were washed with distilled water and kept in the oven at 37 °C for drying. Slides were examined with a fluorescence microscope (Olympus, AX70, Japan) with a specific filter for cyanine fluorochrome. Samples were considered positive for Mhp when the fluorescence emission was visualized in the cilia region, present on the surface of the bronchial epithelium, bronchioles and in the exudate present in the lumen of the respiratory tract.

#### Reverse transcription-polymerase chain reaction

We performed mRNA detection using reverse transcription-polymerase chain reaction (RT-PCR) with the amplification of Mhp glyceraldehyde-3 phosphate dehydrogenase (GAPDH) as a target. Lung samples were subjected to RNA extraction using Trizol (Ambion™) following the manufacturer’s recommendations. RNA was treated with TURBO DNA-free kit (Ambion™). We used RNA, random primer, deoxyribonucleotides, and M-MLV reverse transcriptase (Invitrogen™) for first strand cDNA synthesis.

RT-PCR reactions were performed using a Go Taq Green Master Mix 2x (Promega) and primers for the Mhp GAPDH gene were designed (5′-GTATGATTCCGCCCATGGAAAG-3′ and 5′-CCATGTGGAGCATCCTGTAATC-3′) that amplified a 450 bp fragment. RT-PCR reactions were performed under the following conditions: 94 °C for 2 min, 40 cycles of 94 °C for 45 s, 61 °C for 45 s, 72 °C for 45 s, and a final extension at 72 °C for 7 min. RT-PCR products were analyzed using 1% agarose gel electrophoresis and a transilluminator.

### Genetic characterization

#### PCR based characterization

We selected 16 important genes of Mhp as targets for PCR: 12 genes were expressed in proteins related to virulence and/or immune response, and four genes were expressed in hypothetical proteins with predicted functions. Primers for the 16 target genes were designed based on several genomic sequences of Mhp available in GenBank, and using PrimerQuest Tool (Integrated DNA Technologies). Table 1 shows information about the genes, including the function, classification, primer sequences, annealing temperature, and amplicon size. PCR reactions of the genes were performed under the following conditions: 95 °C for 5 min, 35 cycles of 94 °C for 20 s, annealing with the respective temperature of each pair of primers for 30 s, 72 °C for 45 s, and finally extension at 72 °C for 7 min. We used Mhp strain J as a control for PCR standardization.

**Table 1:** Genes that encode proteins involved in the pathogenesis mechanism and/or immune response; these were used as a molecular target for the PCR assay. The function, classification, nucleotide position and sequences of the forward and reverse primers, annealing temperature and amplicon size (in base pairs) of each target gene are described

Gene	Function	Classification	Nucleotide position	Primer sequences	Annealing temperature (°C)	Amplicon (bp)
*mhp 0107*	P102-like protein	Surface proteins	67	5′-AGTTTATCAGCCGCTGTTGG-3’	58.5	238
			305	5′-GCCCGAACTAAGTCATACCG-3’		
*mhp 0272*	P97-like protein		1627	5′-GTTCGGGCTTGATCTGGTAA-3’	56.6	271
			1898	5′-TGCGAAGCAGTCCGATTT-3’		
*P97*	Surface adhesin		2437	5′-CCAGCAGCTAAACCAGTAGC-3’	55	761
			3198	5′-AGGATCACCGGATTTTGAATC −3’		
*mhp0199*	Protein P102		1581	5′-TAGCCCCACACAACCGAAAA-3’	58	567
			2148	5′-ACCTTGATCAGTTTGAATTGCATCT-3’		
*mhp 0099*	Lipoprotein P95		1297	5′-CCGCTTGATAGCGAGAAGAAA-3’	60	402
			1699	5′-GACGGCTTTGACTACCATCTT-3’		
*mhp 0580*	Membrane nuclease lipoprotein		311	5′-TTGGCTTAGCGGAGATAACG-3’	60	237
			548	5′-AGCGGTCTGGCTCATTCTAA-3’		
*mhp 461*	Multifunctional leucine aminopeptidase on the surface		299	5′-TCTTCTCGCTAAGAGCCGAT-3’	60	400
			699	5′-ACCACCTGAATCAAAAGTAATTCCT-3’		
*mhp 0360*	P37-like ABC transporter substrate-binding lipoprotein		540	5′-TTTACGCCAATCAGCTAGGG-3’	60	210
			750	5′-GGCTTTCTTGATCTCCTCTCG-3’		
*P46*	Membrane surface protein		325	5′-AGTCCAGCGCCAAAAGGATT-3’	57	503
			828	5′-ACCACCTGCTGGATCTTTGT-3’		
*mhp 0067*	Chaperone protein dnaK	Non-surface proteins	27	5′-CGACCTTGGAACAACAAACTC-3’	60	538
			565	5′-CGAAGGTTCCACCGGATAAT-3’		
*nrdF*	Ribonucleotide reductase R2 subunit		691	5′-AGCCCGGAAAAACAAGACGA-3’	60	304
			995	5′-GTTTCTTCAGCAAGCGCCAT-3’		
*mhp372*	Lipoprotein		1562	5′-TGCAAAAAGTTGGTGCAGTT-3’	58	579
			2141	5′-AGACTTATTCCACCTTCGGCT-3’		
*mhp 0443*	Hypothetical protein	Hypothetical proteins	1667	5′-GAGCATCAGGTTCTGGGGTA-3’	60	222
			1889	5′-GAAGGTTGATCCTCGCTCTG-3’		
*mhp 0660*	Lipoprotein		345	5′-GCAGCCCGAAATAACTAGTCC-3’	58.5	283
			628	5′-TTCGTGCGTTAGCAACCTG-3’		
*mhp0418*	Lipoprotein		369	5′-TGCTAGTTTTATTCCAAGCCCT-3’	55.8	599
			968	5′-CTTTGCACGCGCTGGATTAG-3’		
*mhp 0487*	Putative Mg^2+^ transporter		775	5′-GCAGGAATTTCACCTCAGGA-3’	56.6	283
			1058	5′-AGCGAAATTGCCCTGACA-3’

**Table 2:** Frequency of detection of the 16 studied genes, function, classification and locus_tag from Zona da Mata of Minas Gerais and Alto Paranaíba regions

Genes	Function	Classification	Locus_tag	Gene Frequency (%)		
				Alto Paranaíba	Zona da Mata of Minas Gerais	Total
*mhp0107*	P102-like protein	Surface proteins	MHP7448_RS00605	97.5	100	98.4 (63/64)
*mhp0272*	P97-like protein		MHP7448_RS01470	47.5	87.5*	62.5 (40/64)
*P97*	Surface adhesin		MHP7448_RS01085	77.5	62.5	71.9 (46/64)
*mhp0199*	Protein P102		MHP7448_RS01090	32.5	20.8	28.1 (18/64)
*mhp0099*	Lipoprotein P95		MHP7448_RS00565	82.5	91.7	85.9 (55/64)
*mhp0580*	Membrane nucleasse lipoprotein		MHP7448_RS03135	82.5	100	89.1 (57/64)
*mhp461*	Multifunctional leucine aminopeptidase on the surface		MHP7448_RS02520	95	87.5	92.2 (59/64)
*mhp0360*	P37-like ABC transporter substrate-binding lipoprotein		MHP7448_RS01990	65	66.7	65.6 (42/64)
*P46*	Membrane surface protein		MHP7448_RS02785	87.5	70.8	81.3 (52/64)
*mhp0067*	Chaperone protein dnaK	Non-surface proteins	MHP7448_RS00350	90	100	85.9 (55/64)
*nrdF*	Ribonucleotide reductase R2 subunit		MHP7448_0223	90	87.5	89.1 (57/64)
*mhp372*	Lipoprotein		MHP7448_RS02050	77.5	70.8	75 (48/64)
*mhp0443*	Hypothetical protein	Hypothetical proteins	MHP7448_RS02430	92.5	95.8	93.8 (60/64)
*mhp0660*	Lipoprotein		MHP7448_RS03560	100	95.8	98.4 (63/64)
*mhp0418*	Lipoprotein		MHP7448_RS02290	60*	29.2	48.4 (31/64)
*mhp0487*	Putative Mg^2+^ transporter		MHP7448_RS02650	87.5	100	92.2 (59/64)

The asterisk (*) denotes a statistically significant difference (*p* < 0.05; Chi-square test) between the regions

**Table 3:** Mhp-positive samples randomly selected to be partially sequenced and the percentage of similarity between the 16 target genes of Mhp field strains, compared to other Mhp sequences deposited into GenBank (AE017332.1; AE017244.1; AE017243.1; CP003802.1; CP002274.1; CP003131.1)

Gene	Function	Classification	Samples							
			1	2	3	4	5	6	7	8
*mhp 0107*	P102-like protein	Surface proteins	98–100%	99%	99–100%	99%	98–99%	–	–	–
*mhp 0272*	P97-like protein		ND	99%	99%	–	–	–	–	–
*P97*	Surface adhesin		–	–	–	–	–	ND	99%	99%
*mhp0199*	Protein P102		–	–	–	–	–	99–100%	99–100%	99%
*mhp 0099*	Lipoprotein P95		ND	98–99%	–	ND	98–99%	–	–	–
*mhp 0580*	Membrane nucleasse lipoprotein		99–100%	ND	ND	99%	99%	–	–	–
*mhp 461*	Multifunctional leucine aminopeptidase on the surface		–	–	–	–	–	99–100%	98–99%	ND
*mhp 0360*	P37-like ABC transporter substrate-binding lipoprotein		99–100%	–	–	–	99%	–	–	–
*P46*	Membrane surface protein		–	–	–	–	–	99%	99%	ND
*mhp 0067*	Chaperone protein dnaK	Non-surface proteins	99%	99%	99–100%	99%	99%	–	–	–
*nrdF*	Ribonucleotide reductase R2 subunit		–	–	–	–	–	99–100%	ND	99–100%
*mhp372*	Lipoprotein		–	–	–	–	–	ND	ND	98–99%
*mhp 0443*	Hypothetical protein	Hypothetical proteins	98–99%	ND	98–99%	98–99%	98–99%	–	–	–
*mhp 0660*	Lipoprotein		99%	99%	99%	99%	99%	–	–	–
*mhp0418*	Lipoprotein		–	–	–	–	–	98–100%	99–100%	99–100%
*mhp 0487*	Putative Mg^2+^ transporter		99%	98–99%	99%	98–99%	98–99%	–	–	–

*ND* not determined by sequencing, Mhp field strain did not contain the gene

#### Sanger sequencing

Mhp-positive samples were randomly selected to sequence via Sanger partial sequencing. PCR products were purified using a GFX™ PCR DNA and Gel Band Purification Kit (GE Healthcare Illustra™) and sent to be sequenced. Sequences were aligned using DNAMAN 9 (Lynnon Corporation) and CLC genomics software and compared with several Mhp sequences available in GenBank using BLAST.

### Bioinformatic and statistical analysis

Bioinformatic analyses were conducted using BioNumerics 6.6 software (Applied Maths NV, Sint-Martens-Latem, Belgium). A dendrogram was created and the number of clusters was determined based on the following similarity parameters: Dice’s coefficient, 85% similarity cutoff value, and unweighted pair group method using arithmetic averages (UPGMA).

Statistical analyses were performed to compare the lesion score distribution across the regions, including a Fisher’s exact test and Yates’ correction using GraphPad Prism version 7.00 for Windows, GraphPad Software, La Jolla California USA, www.graphpad.com. Statistical significance to check differences in the prevalence of the selected genes in the regions was calculated using a Chi-squared test in R software, and *p* < 0.05 was considered significant.

## Supplementary information

**Additional file 1. Table S1.** Identification of the farm, city, region, and the number of samples collected in both regions studied.

**Additional file 2. Table S2.** Classification of lesion scores of the samples from both regions.

## Data Availability

The datasets used and/or analyzed during the current study are available from the corresponding author on reasonable request. All data generated or analyzed during this study are included in this published article and its supplementary information files.

## Abbreviations

BALT: Bronchus-associated lymphoid tissue
EP: Enzootic pneumonia
FISH: Fluorescence in situ hybridization
GAPDH: Glyceraldehyde-3 phosphate dehydrogenase
HE: Hematoxylin and eosin
Mhp: *Mycoplasma hyopneumoniae*
MLVA: Multiple locus variable-number tandem repeat analysis
PCR: Polymerase chain reaction
RT-PCR: Reverse transcription-polymerase chain reaction
UPGMA: Unweighted pair group method using arithmetic averages
