# Correction to: Genetic variation of Mycoplasma hyopneumoniae from Brazilian field samples

**DOI:** 10.1186/s12866-019-1637-x

**Published:** 2019-11-20

**Authors:** Viviane Sisdelli Assao, Thalita Moreira Scatamburlo, Elaine Nery Araujo, Marcus Rebouças Santos, Carlos Eduardo Real Pereira, Roberto Maurício Carvalho Guedes, Gustavo Costa Bressan, Juliana Lopes Rangel Fietto, Yung-Fu Chang, Maria Aparecida Scatamburlo Moreira, Abelardo Silva-Júnior

**Affiliations:** 1 Federal University of Viçosa, Viçosa, Minas Gerais Brazil; 2 Federal University of Minas Gerais, Belo Horizonte, Minas Gerais Brazil; 3 Cornell University College of Veterinary Medicine, Ithaca, USA

**Correction to: BMC Microbiol**

**https://doi.org/10.1186/s12866-019-1603-7**

Following publication of the original article [1], we have been notified that two of the author names were incomplete or incorrect.

Originally published author names:

- Thalita M Scatamburlo
- Maria Aparecida Scatambulo Moreira

Correct author names:

- Thalita Moreira Scatamburlo
- Maria Aparecida Scatamburlo Moreira

The original article has been corrected.

## References

[1] Assao (2019). Genetic variation of Mycoplasma hyopneumoniae from Brazilian field samples. BMC Microbiol.

