# Supplementary material for: Genetic variation of Mycoplasma hyopneumoniae from Brazilian field samples
Source: BMC Microbiol. 2019 Oct 28;19:234. doi: 10.1186/s12866-019-1603-7 (PMC6819545; doi:10.1186/s12866-019-1603-7)
Supplement: Supplementary file 1 — Additional file 1. Table S1. Identification of the farm, city, region, and the number of samples collected in both regions studied. [file 12866_2019_1603_MOESM1_ESM.docx]

**Supplementary Material**

Table S1: Identification of the farm, city, region, and the number of samples collected in both regions studied.

| Farm | City | Region | Number of samples |
| --- | --- | --- | --- |
| A | Coromandel | Alto Paranaíba | 18 |
| B | Jequeri | Zona da Mata of Minas Gerais | 25 |
| C | Patrocínio | Alto Paranaíba | 29 |
| D | Patrocínio | Alto Paranaíba | 19 |
| E | Patrocínio | Alto Paranaíba | 25 |
| F | Ponte Nova | Zona da Mata of Minas Gerais | 14 |
| G | Ponte Nova | Zona da Mata of Minas Gerais | 28 |
| H | Ponte Nova | Zona da Mata of Minas Gerais | 21 |
| I | Urucânia | Zona da Mata of Minas Gerais | 23 |
| J | Urucânia | Zona da Mata of Minas Gerais | 25 |
| K | Urucânia | Zona da Mata of Minas Gerais | 19 |
| L | Varjão de Minas | Alto Paranaíba | 20 |
