# Supplementary material for: Genetic variation of Mycoplasma hyopneumoniae from Brazilian field samples
Source: BMC Microbiol. 2019 Oct 28;19:234. doi: 10.1186/s12866-019-1603-7 (PMC6819545; doi:10.1186/s12866-019-1603-7)
Supplement: Supplementary file 2 — Additional file 2. Table S2. Classification of lesion scores of the samples from both regions. [file 12866_2019_1603_MOESM2_ESM.docx]

**Supplementary Material**

Table S2: Classification of lesion scores of the samples from both regions

| Region | Lesion Score  (number of samples) | | | |
| --- | --- | --- | --- | --- |
|  | **1** | **2** | **3** | **4** |
| Zona da Mata of Minas Gerais | 8.33%  (2/24) | 33.33%  (8/24) | 37.5%  (9/24) | 20.93%  (5/24) |
| Alto Paranaíba | 15%  (6/40) | 60%  (24/40) | 20%  (8/40) | 5%  (2/40) |
